# Rhino-Orbital Mucormycosis in a Postpartum Diabetic Patient: An Atypical Presentation of Oral Ulcers and Facial Swelling

**DOI:** 10.7759/cureus.81058

**Published:** 2025-03-23

**Authors:** Taha Zahid Chaudhry, Muhammad Abubakar, FNU Manisha

**Affiliations:** 1 Internal Medicine, Holy Family Hospital, Rawalpindi, Rawalpindi, PAK; 2 Internal Medicine, Wah Medical College, Wah Cantonment, PAK; 3 Internal Medicine, Peoples University of Medical and Health Sciences For Women, Nawabshah, PAK

**Keywords:** antifungal therapy, case report, diabetes mellitus, invasive fungal infection, mucormycosis, postpartum immunosuppression, rhino-orbital mucormycosis, surgical debridement

## Abstract

Mucormycosis is a rare, life-threatening fungal infection primarily affecting immunocompromised individuals, particularly those with poorly controlled diabetes mellitus. This case report presents a 40-year-old female with a decade-long history of uncontrolled type 2 diabetes who developed progressively worsening oral ulcers, facial swelling, and vomiting, following a recent stillbirth. The patient exhibited extensive necrotic lesions involving the nose, oropharynx, and soft palate, leading to a high suspicion of invasive fungal infection. Diagnostic imaging with magnetic resonance imaging (MRI) revealed extensive sinus and orbital involvement, while histopathological examination confirmed rhino-orbital mucormycosis. The patient was managed with liposomal amphotericin B, strict glycemic control, and surgical debridement to remove necrotic tissue. This case underscores the importance of early recognition and multidisciplinary management of mucormycosis, particularly in atypical presentations involving postpartum immunosuppression. Given the rising incidence of invasive fungal infections, clinicians must maintain a high index of suspicion in high-risk patients, especially those with uncontrolled diabetes. Awareness of mucormycosis and its varied clinical presentations can aid in timely diagnosis and improve patient outcomes. The report highlights a unique presentation in a postpartum female, suggesting a potential link between obstetric complications and invasive fungal infections, warranting further research into postpartum-related immunosuppression as a risk factor.

## Introduction

Mucormycosis is a rare but aggressive fungal infection caused by fungi of the order Mucorales, primarily affecting immunocompromised individuals. It most commonly manifests as rhino-orbital-cerebral disease in patients with uncontrolled diabetes mellitus, hematological malignancies, or those undergoing immunosuppressive therapy [1]. The hallmark of the disease is angioinvasion, which leads to vascular occlusion, tissue necrosis, and rapid progression if left untreated [2]. Global incidence rates have risen significantly, particularly in regions with a high prevalence of poorly controlled diabetes, where hyperglycemia and ketoacidosis create a favorable environment for fungal proliferation [3]. Despite its increasing recognition, atypical presentations such as oral ulcers or isolated facial swelling remain underreported, often leading to diagnostic delays.

While diabetes mellitus is a well-established predisposing factor, postpartum immunosuppression is a relatively unexplored risk factor for invasive fungal infections. Pregnancy-induced immunological changes, coupled with the physiological stress of delivery or obstetric complications such as stillbirth, may transiently impair cellular immunity, increasing susceptibility to opportunistic infections [4]. Although few reports in the literature have described mucormycosis in postpartum patients, these cases highlight the need for increased awareness of the potential interplay between obstetric immunosuppression and other comorbidities such as diabetes. Recognizing this connection is essential for early diagnosis and intervention, particularly in regions with limited access to advanced healthcare facilities.

This case report discusses an unusual presentation of rhino-orbital mucormycosis in a postpartum female with poorly controlled diabetes, marked by progressive oral ulcers and facial swelling. It emphasizes the importance of early clinical suspicion, multidisciplinary management, and the consideration of postpartum immunosuppression as a contributing factor. By addressing the diagnostic challenges and treatment strategies, this report aims to provide clinicians with practical insights to improve the timely recognition and management of mucormycosis, particularly in high-risk populations.

## Case presentation

A 40-year-old female, known to have poorly controlled type 2 diabetes mellitus for the past decade, presented with a 15-day history of progressively worsening oral ulcers accompanied by facial swelling and a three-day history of persistent, non-bloody vomiting. The patient reported that the ulcers initially appeared painless but gradually increased in size and number, becoming painful and involving the oropharyngeal region. These ulcers were irregular in shape, yellowish in appearance, and surrounded by disrupted mucosa. Over time, the lesions spread to the nasopharynx, resulting in increasing facial swelling and nasal obstruction. She also experienced mouthful, non-projectile vomiting containing food particles, which was not associated with fever, abdominal pain, or headache. Of note, the patient had suffered a stillbirth one month prior to presentation, a factor that may have contributed to her compromised immune status. Despite her longstanding diabetes diagnosis, she had poor compliance with oral hypoglycemic medications, and her blood sugar levels were inadequately controlled, with a recent HbA1c level of 10.8% indicating chronic hyperglycemia. A significant family history of diabetes was noted, though no similar presentations were reported in her immediate relatives.

On clinical examination, the patient exhibited extensive blackened, necrotic ulcers involving the nose, oropharynx, and soft palate, indicative of tissue ischemia and necrosis. There was evidence of nasal crusting and significant soft tissue swelling around the facial and periorbital regions, leading to partial nasal airway obstruction and dyspnea. The extent and necrotic appearance of the lesions raised a high index of suspicion for invasive fungal infections, particularly mucormycosis, given her immunocompromised state. Differential diagnoses included Wegener’s granulomatosis due to the necrotic lesions and potential sinonasal involvement. Given the aggressive clinical progression and the patient's significant risk factors, an urgent diagnostic workup for mucormycosis was initiated.

The baseline investigations, summarized in Table 1, revealed significant abnormalities indicative of an underlying infectious process and metabolic derangements associated with poorly controlled diabetes. The patient exhibited severe anemia with microcytic and hypochromic indices, suggestive of iron deficiency anemia, likely due to chronic malnutrition and poor socioeconomic conditions. There was thrombocytosis, indicating a reactive process or underlying inflammation. Renal function was compromised, with impaired renal clearance likely secondary to diabetic nephropathy. Elevated alkaline phosphatase (ALP) suggested possible bone turnover or tissue damage, while glucosuria was consistent with uncontrolled diabetes. These findings necessitated further imaging and diagnostic tests to confirm the underlying pathology and guide management.

**Table 1:** Summary of the initial blood test results. MCV: Mean corpuscular volume; MCH: Mean corpuscular hemoglobin; ALP: Alkaline phosphatase; WBC count: White blood cell count

Investigation	Result	Reference Range
Hemoglobin (Hb)	7.62 g/dL	13-18 g/dL
MCV	64.32 fL	77-95 fL
MCH	19.62 pg	26-32 pg
Platelet Count	682x10^9/L	150-400x10^9/L
Serum Creatinine	1.82 mg/dL	0.5-0.9 mg/dL
ALP	254 U/L	40-120 U/L
Urine Glucose	1+	Negative
WBC Count	8.6x10^9/L	4-11x10^9/L
Neutrophils	70%	40-80%

**Figure 1:**
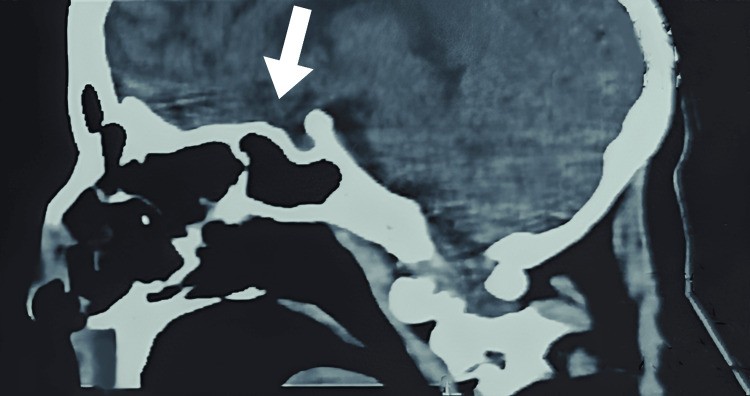
Computed tomography of the skull and orbits shows extensive sinus involvement, as pointed by the arrow.

Computed tomography (CT) of the skull and orbits showed extensive sinus involvement, with significant opacification and mucosal thickening in the maxillary, ethmoid, and sphenoid sinuses. Orbital involvement was evident, with changes suggesting the spread of infection into the eye regions. Bone erosion was visible in the sinus walls and hard palate, with associated soft tissue swelling in the facial and orbital regions. The nasal cavity showed mucosal thickening with necrotic changes. No large brain lesions were noted, and vascular structures appeared intact. The imaging findings, as given in Figure 1, confirmed extensive rhino-orbital mucormycosis with aggressive tissue invasion, prompting the immediate initiation of antifungal therapy.

**Figure 2:**
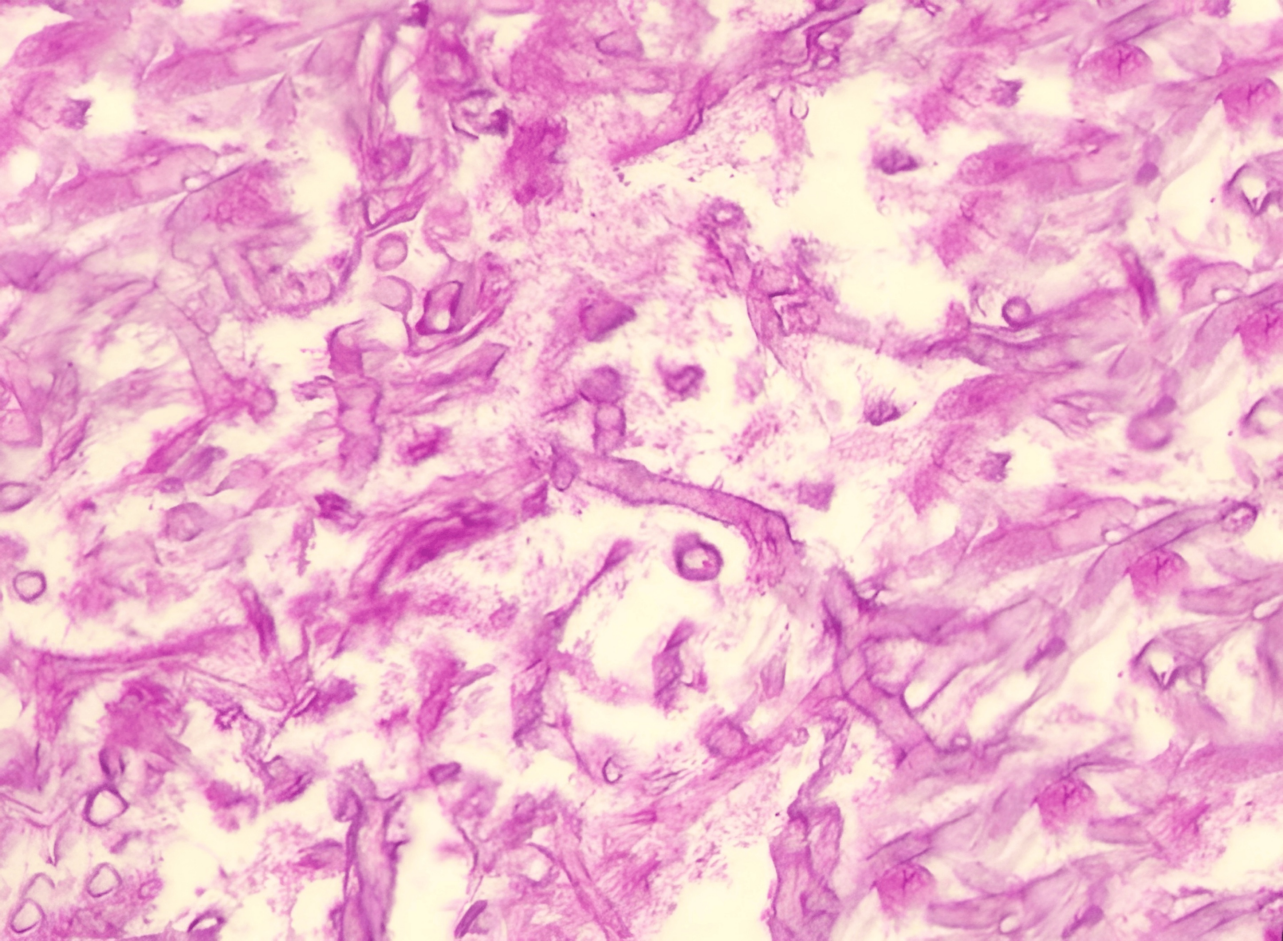
Histopathology reveals broad, irregular, ribbon-like fungal hyphae branching at wide angles.

Histopathological examination of the debrided tissue confirmed the diagnosis of mucormycosis. The samples were obtained during surgical debridement of necrotic tissue involving the nasal cavity, oropharynx, and orbit, which was performed by the otorhinolaryngology (ENT) team in coordination with maxillofacial surgeons. Microscopy revealed broad, irregular, ribbon-like fungal hyphae branching at wide angles (~90 degrees) with angioinvasion, a hallmark feature of mucormycosis. Extensive necrosis was observed, with inflammatory infiltration by neutrophils and macrophages. Granulomatous reactions were also noted. Gomori methenamine silver (GMS) staining was positive, confirming the presence of Mucorales fungi. Culture results yielded the growth of *Mucor* species, further solidifying the diagnosis. The combination of clinical, radiological, and histopathological findings confirmed rhino-orbital mucormycosis in this immunocompromised diabetic patient, necessitating aggressive antifungal therapy and surgical intervention. The histopathological image is illustrated in Figure 2.

The patient was promptly initiated on liposomal amphotericin B, the first-line antifungal therapy for mucormycosis, at a dose of 5 mg/kg/day. Strict glycemic control was enforced through an intravenous basal-bolus insulin regimen, with frequent capillary glucose monitoring. At the time of admission, her random blood glucose was 356 mg/dL, and following the initiation of insulin therapy, levels gradually stabilized within the range of 140-180 mg/dL. Supportive care included empiric broad-spectrum antibiotics, specifically piperacillin-tazobactam and vancomycin, to prevent secondary bacterial infections. Additionally, iron supplementation and blood transfusions were administered to address her severe anemia. Given the extent of tissue necrosis and disease spread, surgical debridement was performed to remove the necrotic tissue from the nasal cavity, oropharynx, and orbit, minimizing the fungal load and preventing further angioinvasion. Histopathological examination of the debrided tissue confirmed the diagnosis of mucormycosis. Postoperatively, the patient was closely monitored in a sterile environment to reduce the risk of nosocomial infections, with ongoing renal function monitoring due to the potential nephrotoxic effects of amphotericin B.

The patient's clinical condition stabilized after a few days of treatment. Facial swelling reduced, and her breathing improved significantly. Over the following weeks, the patient showed signs of favorable response to treatment, including decreased sinus involvement and soft tissue inflammation. Despite the initial improvement, the patient's prognosis remained guarded due to the high mortality rate associated with rhino-orbital mucormycosis and her underlying immunocompromised state. Long-term follow-up included continuation of antifungal therapy for six to eight weeks, glycemic control through a structured insulin regimen, and regular ENT and ophthalmology evaluations to monitor for recurrence or residual disease.

Family members were counseled extensively regarding the disease's aggressive nature, the importance of diabetes management, and the need for early recognition of symptoms in the future. The case highlights the critical importance of a multidisciplinary approach involving infectious disease specialists, endocrinologists, ENT surgeons, and ophthalmologists to improve patient outcomes in such life-threatening fungal infections.

## Discussion

Rhino-orbital mucormycosis is a rare but aggressive fungal infection commonly seen in older males with underlying conditions such as uncontrolled diabetes, hematological malignancies, or immunosuppressive therapy [3]. The estimated global incidence of mucormycosis ranges from 0.005 to 1.7 per million population annually, but is significantly higher in countries like India, with reported rates of 14 per 100,000 population, largely due to the high burden of uncontrolled diabetes [3,4]. This case highlights a unique presentation in a younger postpartum female with poorly controlled type 2 diabetes and recent immunosuppression following a stillbirth. The disease progression from painless oral ulcers to extensive facial swelling and necrotic lesions is uncommon, setting this case apart from typical presentations of rapid sinonasal involvement. A targeted literature review revealed very few reported cases of rhino-orbital mucormycosis in postpartum women and even fewer with initial symptoms dominated by oral ulceration and facial swelling. Most documented cases in postpartum settings have focused on sinus or orbital involvement, with minimal mention of oropharyngeal manifestations. This suggests that the combination of postpartum immunosuppression and diabetes with atypical oral involvement may represent an underrecognized clinical pattern deserving further investigation.

The pathophysiology of mucormycosis involves angioinvasion, leading to vascular occlusion, tissue ischemia, and necrosis. Uncontrolled diabetes remains a major predisposing factor due to hyperglycemia, impaired neutrophil function, and elevated free iron levels that promote fungal proliferation [5]. In this case, postpartum immunosuppression likely compounded these risks, accelerating disease progression. Obstetric complications, such as stillbirth, may transiently impair immune function, rendering patients more susceptible to invasive fungal infections. Given the patient’s presentation with oral ulcers, facial swelling, and necrotic lesions, the differential diagnoses initially included necrotizing fasciitis, bacterial sinusitis with orbital cellulitis, granulomatosis with polyangiitis (Wegener’s granulomatosis), and malignancies such as squamous cell carcinoma. However, the rapid progression, extensive tissue necrosis, and hallmark findings on histopathology supported the diagnosis of mucormycosis. This highlights the need for increased vigilance in postpartum patients with comorbidities such as diabetes, especially when clinical features are atypical or mimic other aggressive pathologies.

The diagnostic challenges of mucormycosis stem from its nonspecific symptoms, such as oral ulcers and facial swelling, which may mimic other conditions like bacterial sinusitis or autoimmune diseases. In immunocompromised patients, early imaging with MRI and histopathological confirmation through biopsy is critical for timely diagnosis [6]. The detection of characteristic broad (5-20 µm), non-septate or pauci-septate, ribbon-like fungal hyphae that branch at right or obtuse angles (~90°) with angioinvasion is a diagnostic hallmark of Mucorales. These features help distinguish Mucorales from other filamentous fungi such as *Aspergillus*, which typically exhibit thin, septate hyphae with acute angle (45°) branching. Early identification of these unique histopathologic traits is essential to differentiate mucormycosis from other fungal infections and to prevent the rapid progression of this life-threatening disease.

Management of mucormycosis requires a combination of high-dose liposomal amphotericin B and surgical debridement to remove necrotic tissue and reduce fungal load [7]. A multidisciplinary approach involving infectious disease specialists, endocrinologists, and surgeons significantly improves outcomes. However, delayed diagnosis and the aggressive nature of the disease contribute to high mortality rates, which remain as high as 50%. Long-term management, including continued antifungal therapy and strict glycemic control, is crucial to prevent recurrence [8,9].

This case underscores several critical points for clinicians: the importance of high clinical suspicion for mucormycosis in immunocompromised patients, particularly those with diabetes; the need to recognize atypical presentations, such as oral ulcers; and the potential impact of postpartum immunosuppression as a risk factor. Novel insights include the need for further research into pregnancy-related immunosuppression and its role in fungal infections. Prognosis in rhino-orbital mucormycosis remains guarded, with reported mortality rates ranging from 30% to over 50%, depending on the extent of disease and timing of intervention. Delayed diagnosis, intracranial extension, and poor glycemic control are associated with worse outcomes, while early surgical debridement, prompt antifungal therapy, and tight metabolic control significantly improve survival rates. In this case, timely intervention led to initial clinical stabilization, although long-term outcomes remain uncertain due to the aggressive nature of the infection. Awareness, early diagnosis, and a multidisciplinary approach remain the cornerstone of improving survival and functional outcomes in these high-risk patients.

## Conclusions

This case brings attention to an uncommon and underreported presentation of rhino-orbital mucormycosis in a postpartum diabetic female, where oral ulcers and facial swelling were the initial signs rather than the typical sinonasal or orbital symptoms. The interplay between postpartum immunosuppression and poorly controlled diabetes highlights a potential synergistic risk factor for invasive fungal infections. This atypical clinical picture calls for heightened clinical awareness in postpartum patients presenting with unexplained orofacial symptoms, particularly in regions with high diabetes prevalence. Future studies are warranted to explore the immunological changes following obstetric complications, such as stillbirth, and their role in increasing susceptibility to opportunistic infections like mucormycosis. Early identification of such high-risk profiles may aid in the development of targeted screening and management strategies, ultimately improving patient outcomes.

## References

[1] Hernández JL, Buckley CJ (2023). Mucormycosis. StatPearls [Internet].

[2] Barbosa OA, do Amaral ES, Pinheiro Furtado G, Filomeno da Silva VC, de Alencar Isabele M, Aguiar de Freitas K (2024). Fungal necrotizing fasciitis due to mucormycosis following contaminated substance inoculation: a report of two cases. Eur J Case Rep Intern Med.

[3] Elmonofy O, Ghanem M, Abdelwahab M, Mubarak FA (2022). A prospective case series on rhino-orbital cerebral mucormycosis in Egypt: epidemiology, systemic implications, and treatment. Int J Surg Open.

[4] Singh AK, Singh R, Joshi SR, Misra A (2021). Mucormycosis in COVID-19: a systematic review of cases reported worldwide and in India. Diabetes Metab Syndr.

[5] (2024). Mucormycosis (Zygomycosis). https://emedicine.medscape.com/article/222551-overview.

[6] Sharma A, Goel A (2022). Mucormycosis: risk factors, diagnosis, treatments, and challenges during COVID-19 pandemic. Folia Microbiol (Praha).

[7] Beaver R, Garza B, Vallabhaneni H, Cahuayme-Zuniga L, Midturi J, LaDow T (2021). Use of topical amphotericin in a case of refractory sino-orbital angioinvasive mucormycosis. Med Mycol Case Rep.

[8] Yang P, Ju Y, Hu Y, Xie X, Fang B, Lei L (2023). Emerging 3D bioprinting applications in plastic surgery. Biomater Res.

[9] Spellberg B, Edwards J Jr, Ibrahim A (2005). Novel perspectives on mucormycosis: pathophysiology, presentation, and management. Clin Microbiol Rev.

